# Explantation and Simultaneous Explantation-Reimplantation of Spinal Cord Stimulation Paddle Electrodes: Complication Rate and Predisposing Factors

**DOI:** 10.1227/neuprac.0000000000000055

**Published:** 2023-08-18

**Authors:** Xenia Kuparinen, Abdirisak Ahmed Haji Omar, Nuutti Vartiainen, Johan Marjamaa, Joonatan Gröndahl, Riku Kivisaari, Julio Resendiz-Nieves

**Affiliations:** * Doctoral Programme in Clinical Research, University of Helsinki, Helsinki, Finland;; ‡ Department of Neurosurgery, Helsinki University Hospital, Helsinki, Finland

**Keywords:** Complications, Electrode explantation, Electrode removal, Paddle electrode, Spinal cord stimulation, Explantation-reimplantation

## Abstract

**BACKGROUND AND OBJECTIVES:**

Spinal cord stimulation (SCS) is an effective treatment for chronic pain that does not respond to conservative treatment. Nonetheless, up to 38% of all implanted SCS electrodes are explanted, and while the risks involved in the surgical implantation of SCS paddle electrodes are well documented, there is scarce information about SCS explantations and their associated complications. We aimed to document the complication rate and identify their predisposing factors in SCS paddle electrode explantations and simultaneous explantation-reimplantations.

**METHODS:**

We retrospectively reviewed the outcomes and the characteristics of all patients who underwent explantation of surgically implanted SCS paddle electrodes at the Helsinki University Hospital Department of Neurosurgery between February 2005 and October 2020.

**RESULTS:**

One hundred thirty-one explantations were performed on 106 patients. The complication rate was 18.3% (24 operations). Major complications occurred during 5 operations (3.8%). No permanent neurological deficits were recorded. Smoking predisposed patients to postoperative complications (*P* = .023). On average, patients who suffered complications required a day longer hospitalization (2.22 vs 2.92, *P* = .011). Patients who had repeated explantations (3 or more) suffered significantly more complications than patients who had only 1 or 2 operations (62.5% vs 15.4%, *P* = .005).

**CONCLUSION:**

Our results suggest that the explantation of the SCS paddle electrode is a relatively safe surgical procedure. Although severe complications occurred, they were successfully managed. Repeated explantations should be treated cautiously as they seem to increase the complication rate considerably.

**ABBREVIATIONS:**
CRPS: complex regional pain syndrome
DM: diabetes mellitus
SCS: spinal cord stimulation.

Spinal cord stimulation (SCS) is a safe, effective, and reversible treatment for patients suffering from severe chronic pain unresponsive to conservative treatment, such as failed back surgery syndrome, complex regional pain syndrome (CRPS), and painful neuropathy.^1^ Despite good efficacy and optimized patient selection, the explantation rate for all SCS electrodes is up to 38% and for surgically implanted SCS paddle electrodes is up to 25%.^2-4^ The most common reasons for explantation are loss of benefit, infection, need for MRI, and hardware malfunction.^5,6^

Although the risks involved in the implantation of SCS electrodes are well documented, there is scarce information about SCS explantations and their associated complications. We focused our study on surgically implanted SCS paddle electrodes that require an approach through a laminotomy or, in some centers, a laminectomy. Their considerable size and more invasive removal procedure can predispose patients to complications.

Typically for surgically implanted foreign bodies, the SCS electrode eventually becomes encapsulated.^7,8^ The dura below the electrode usually becomes thinner and frailer, which can lead to dural tear and cerebrospinal fluid leakage during explantation. Manipulation of scar tissue and the electrode can cause medullar contusion leading to neurological impairment. Reoperating on already damaged tissue makes hemostasis more challenging, predisposing to epidural hematoma formation and slower wound healing, increasing the risk of infection. Extended bone removal increases the risk of instability. All these risks must be considered and discussed with the patient when considering SCS paddle electrode removal.

In this study, we examined the surgical complications and the associated risk factors involved in the removal of SCS paddle electrodes.

## METHODS

We retrospectively reviewed the characteristics and surgical outcomes of all Helsinki University Hospital Department of Neurosurgery patients who underwent explantation of SCS paddle electrodes between February 2005 and October 2020.

We defined explantation as the removal of a permanently implanted SCS paddle electrode from the epidural space in the cervical or thoracic spine after a minimum of 29 days from permanent implantation. Our series included operations where the electrode was removed and, during the same surgery, reimplanted to a new site or replaced with a new electrode at the same epidural location.

All patient records have been reviewed for a minimum of 6 months postoperatively to ensure proper documentation. Twelve patients were excluded due to poor documentation.

We examined patient characteristics known to affect the surgical outcome during other spinal operations; smoking, which included patients who had quit smoking less than a year ago; body mass index; the American Society of Anesthesiologists (ASA) physical status classification system classes; immunosuppression; and diabetes mellitus (DM).^9-12^

We also examined the effects on the complication rate of the SCS electrode's model and location (thoracic vs cervical) as well as the reason for explantation and time between implantation and explantation. Complication rates of electively performed SCS paddle electrode explantation vs emergency operations were compared. Surgeons' qualifications' effect on complication rate as well as the duration of operation were also analyzed.

Complications were divided into major and minor. Major complications included death, cerebrospinal fluid leak, new neurological deficit, or a complication requiring further surgical intervention. All other complications were considered minor. The complication rate is reported per case unless specified otherwise, meaning that some patients were counted multiple times. We decided to do this because of considerable variability in patients' characteristics between operations.

All statistical analysis was performed using SPSS (IBM, version 25). We used Fisher exact test, independent samples *t*-test, and Mann-Whitney *U* tests to examine differences between groups. All tests were 2-tailed, with alpha set to .05. Strengthening the Reporting of Observational Studies in Epidemiology (STROBE) reporting guideline was implemented. Our research has been approved by the Ethics committee of the University of Helsinki and follows the principles outlined in the Declaration of Helsinki. Individual patient consent was not required because data were deidentified.

## RESULTS

In our department, we performed 448 permanent SCS paddle electrode implantations between the years 2000 and 2020. One hundred six patients underwent 131 explantations of surgically implanted SCS paddle electrodes between 2005 and 2020. 89 patients had SCS paddle electrodes explanted once, 13 patients twice, 2 patients 3 times, and 2 patients 5 times.

Table 1 presents patient demographic. More women (54.5%) than men (45.5%) were explanted. The patient's average age was 49.84 years (SD = 12.4). Most of our patients belonged to ASA classes II and III. Body mass index (28.29 kg/m^2^ vs 28.40 kg/m^2^, *P* = .569) or ASA classification (*P* = .575) did not affect the complication rate.

**TABLE 1.** Patient Demographics^a^

Variable	Total sample	No complications	Complications	*P* value
	n (%) or mean (SD)	n (%) or mean (SD)	n (%) or mean (SD)	
Age and sex	n = 131	n = 107	n = 24	
Age	49.84 (12.4)	50.42 (12.62)	47.25 (11.33)	.24
Male	60 (45.5%)	48 (80.0%)	12 (20.0%)	.658
Female	71 (54.5%)	59 (83.1%)	12 (16.9%)	
	n = 128	n = 106	n = 22	
BMI	28.31 (5.31)	28.29 (5.4)	28.40 (4.80)	.569
ASA class	n = 124	n = 101	n = 23	
ASA class I	9 (7.3%)	8 (88.9%)	1 (11.1%)	.575
ASA class II	71 (57.3%)	60 (84.5%)	11 (15.5%)	
ASA class III	39 (31.5%)	29 (74.4%)	10 (25.6%)	
ASA class IV	5 (4.0%)	4 (80.0%)	1 (20.0%)	
Smoking	n = 110	n = 90	n = 20	.023^b^
Nonsmokers	65 (59.1%)	58 (89.2%)	7 (10.8%)	
Smokers	45 (40.9%)	32 (71.1%)	13 (28.9%)	
Diabetes	n = 121	n = 97	n = 24	.073
No diabetes	101 (83.5%)	84 (83.2%)	17 (16.8%)	
DM type I/II	20 (16.5%)	13 (65.0%)	7 (35.0%)	
Immunodeficiency	n = 129	n = 105	n = 24	.464
No. immunodeficiency	126 (97.7%)	103 (81.7%)	23 (18.3%)	
Immunodeficiency	3 (2.3%)	2 (66.7%)	1 (33.3%)	

BMI, body mass index; ASA Class, American Society of Anesthesiologists Classification; DM, diabetes mellitus.

a Each re-explantation is considered as a new patient due to significant change in patients' age, weight, and ASA class between each operation. Total sample size for different variables differs due to incomplete documentation available.

b Significant result.

In total, 40.9% of the population smoked. Smoking correlated with a higher complication rate (*P* = .023). Suffering from DM type I or II noticeably increased the complication rate. However, this was not statistically significant (16.8% vs 35.0%, *P* = .073).

Table 2 presents the location and the model of electrodes. 35 (26.7%) electrodes were removed from the cervical spine, and 96 (73.3%) electrodes were removed from the thoracic spine. Explantation from the cervical spine had a higher complication rate than explantation from the thoracic spine, although this was not statistically significant (25.7% vs 15.6%, *P* = .207). The electrode model had no impact on the complication rate.

**TABLE 2.** Electrode Location and Model

Variable	Total sample n (%)	No complications n (%)	Complications n (%)	*P* value
Location of electrode	n = 131	n = 107	n = 24	
Cervical	35 (26.7%)	26 (74.3%)	9 (25.7%)	.207
Thoracic	96 (73.3%)	81 (84.4%)	15 (15.6%)	
Type of electrode in cervical spine	n = 35			
Symmix	19 (54.3%)			
Boston scientific 2 × 8	6 (17.1%)			
Specify hinged 2 × 4	3 (8.6%)			
Specify 2 × 4	2 (5.7%)			
Resume TL	1 (2.9%)			
Resume	1 (2.9%)			
Quadripolar plus	1 (2.9%)			
Lamitrode 1 × 8	1 (2.9%)			
Medtronic 2 × 8	1 (2.9%)			
Type of electrode in thoracic spine	n = 96			
Symmix	51 (53.1%)			
St. Jude Penta	14 (14.6%)			
Specify 5-6-5	13 (13.5%)			
Specify hinged 2 × 4	11 (11.5%)			
Resume TL	2 (2.1%)			
St. Jude 2 × 4	2 (2.1%)			
Resume	1 (1.0%)			
Specify 2 × 4	1 (1.0%)			
Quadripolar plus	1 (1.0%)			

The most common reason for explantation was loss of benefit. None of the reasons for explantation correlated with a higher complication rate (Table 3).

**TABLE 3.** Reason for Explantation

Variable	Total sample n (%) or mean (SD)	Complications n (%) or mean (SD)	*P* value
	n = 132	n = 24	
Loss of benefit	58 (44.3%)	12 (20.7%)	.546
Infection	28 (21.4%)	4 (13.8%)	.866
Need for MRI	21 (16.0%)	1 (4.8%)	.12
Malfunction of electrode	20 (15.3%)	5 (25.0%)	.742
Migration	4 (3.1%)	2 (50.0%)	.284

When the reason for the explant was loss of benefit or malfunction, a considerable number of patients had their electrodes replaced with a new one or relocated to a new site in the same operation. There was no significant difference in the complication rate between those having their electrode removed and those having their electrode removed and replaced with a new one or the old one relocated (16.5% vs 21.2%, *P* = .499) (Table 4).

**TABLE 4.** Replacement/Relocation vs Removal

Variable	Total sample n (%)	No complications n (%)	Complications n (%)	*P* value
Operation type	n = 131	n = 107	n = 24	
Replacement or relocation of the electrode^a^	52 (39.7%)	41 (78.8%)	11 (21.2%)	.499
Removal of the electrode	79 (60.3%)	66 (83.5%)	13 (16.5%)	

a During the explantation operation.

Postoperative complications occurred during 24 operations (18.3%) (Table 5). In 7 of these operations, patients suffered more than 1 complication (5.3%). Major complications occurred during 5 operations (3.8%) and are described below.

**FIGURE 1.**
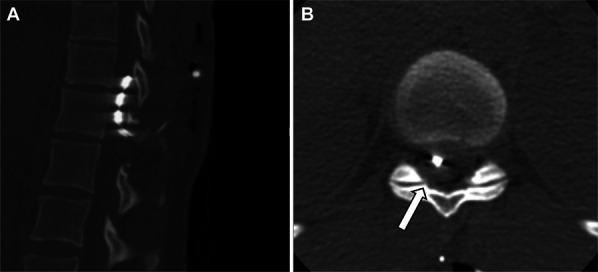
**A**, and **B**, CT of the patient presenting with paraparesis after SCS electrode explantation and replacement. **B**, The arrow is pointing at an epidural hematoma on top of the electrode compressing the spinal cord. This figure is original to this submission, so no credit or license is needed.

1. Patient's electrode was explanted and replaced with a new one due to lack of coverage. Just hours after the operation, the patient developed progressive paraparesis. Emergency thoracic computed tomography (CT) showed an epidural hematoma on top of the electrode (Figure 1). Emergency evacuation of hematoma was performed. The patient made a full recovery.
2. Patient's electrode was explanted due to wire breakage and replaced by a new electrode. A few hours after the operation, the patient developed progressive paraparesis. Emergency CT imaging did not show any etiological explanation. An emergency exploratory operation was performed, during which no hematoma or other explanation for symptoms was detected. Nonetheless, it was decided to remove the electrode from the epidural space. Postoperatively, patient's symptoms resolved. A month later, the patient developed drooping of both feet and severe worsening of old neuropathic pain. MRI did not show any explanation for the symptoms. Psychiatric intervention in the form of therapy and medication as well as optimization of pain medication resolved these issues.
3. The patient had the SCS electrode removed due to infection. Two weeks after the explantation, the patient developed wound dehiscence with deep abscess formation, which was revised in the operating theater. Bacterial cultures showed cefalexin-resistant *Staphylococcus epidermis*, which was treated with antibiotics. After discharge, the patient suffered prolonged incision site pain and had to be readmitted for pain treatment for 4 days.
4. The patient had the SCS paddle electrode explanted due to infection. Postoperatively the patient developed an abscess due to *Staphylococcus aureus* requiring wound revision under general anesthesia. After the revision, the patient developed a subcutaneous seroma, which was punctured several times but, despite that, required a new revision under general anesthesia. After the second revision, the patient developed a new subcutaneous hematoma, which was surgically evacuated. After the evacuation of the subcutaneous hematoma, the patient developed another subcutaneous seroma that was punctured 3 times, after which the symptoms resolved.
5. SCS electrode was explanted due to malfunction of the electrode. The explantation was followed by immediate replacement with a new electrode. Postoperatively, the patient developed a painful subcutaneous hematoma that converted into a seroma, followed by a surgical site infection with cefalexin-resistant *Staphylococcus epidermis*. It was decided to remove the electrode and all other foreign material, followed by antibiotic treatment.

**TABLE 5.** Complications

Variable	Total sample n (%)^a^
Major complications	n = 5
Paraparesis	2 (40%)
Infection with abscess formation	2 (40%)
Subcutaneous hematoma/seroma	1 (20%)
Minor complications	n = 24
Subcutaneous hematoma/seroma	7 (29.2%)
Infection	6 (25%)
Wound dehiscence	5 (20.8%)
Functional limb clumsiness and paresthesia	3 (12.5%)
Abdominal pain	2 (8.3%)
Paresthesia	1 (4.2%)

a Total of 29 complications occurred during 24 operations.

A total of 24 minor complications occurred during 19 operations (14.5%). Postoperatively, 6 patients suffered from a clinically diagnosed superficial wound infection and were treated with antibiotics. Bacterial swabs were taken in 3 out of 6 cases. Two swabs showed *Staphylococcus epidermis*, and one came back negative. Besides antibiotic treatment, no other intervention was required. Seven patients suffered subcutaneous hematoma or seroma and 5 suffered wound dehiscence, all of which resolved spontaneously.

Two patients reported feeling of clumsiness of the left upper limb after the electrode was removed, in one case from the cervical spine and the other from the thoracic spine; during the clinical examination, no clumsiness or motoric weakness was evident and radiological imaging (MRI for former and CT for latter case) did not reveal any explanation for the symptoms. The symptoms persisted at the last follow-ups at 81 and 949 days, respectively. After the explantation of a thoracic electrode, 1 patient experienced foot drop sensation of the left foot and another paresthesia and pain sensations in the left lower limb. MRI imaging did not reveal any explanation for symptoms. In both cases, symptoms resolved in 7 days. Two patients suffered from acute abdominal pain unrelated to the implantable pulse generator location and had no etiological findings on CT. One of the patients had to be hospitalized for pain treatment (Table 5).

**FIGURE 2.**
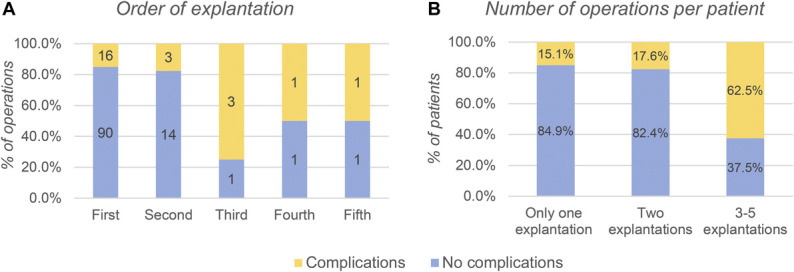
The total number of explantations—131. The total number of patients—106. **A**, The complication rate of each explantation. Here, we can see that after the second explantation, the complication rate significantly rises. **B**, The cumulative number of complications per patient depending on how many operations they had in total. If a patient had more than 2 explantations, the risk of suffering a complication at some point significantly increased. This figure is original to this submission, so no credit or license is needed.

Figure 2 shows that the complication rate rises with each reoperation, especially after the second explantation. The complication rate in the first explantation was 15.1%; after the second explantation, the complication rate was 17.6%, and if 3 to 5 explantations were performed, the complication rate was 62.5%. When explantation was performed for the third, fourth, or fifth time, 5 out of 8 operations led to complications. Patients who underwent 1 or 2 explantation operations suffered significantly fewer complications than patients who underwent explantation operation 3 to 5 times (15.4% vs 62.5%, *P* = .005).

Two patients underwent 5 explantation procedures. The first patient was implanted due to chronic degenerative disk disease pain; the patient was explanted twice due to a change in stimulation sensation and thrice due to hardware malfunction. Operations were performed over an 11-year period. At the last explantation, the electrode was simultaneously replaced, and the patient continues to benefit from the stimulation. The second patient received SCS stimulator first time for chronic degenerative disk disease pain; after the permanent electrode was implanted, the patient no longer benefited from stimulation and required MRI for further investigation, and thus, the electrode was removed. Seven years later, the patient received a CRPS diagnosis, entered a new SCS stimulation trial, and received a permanent implant. The electrode had to be removed and replaced twice due to electrode migration and once due to wire breakage. Finally, the patient no longer benefitted from SCS therapy, and due to implantable pulse generator site pain, it was decided to remove the whole stimulator.

On average, the SCS paddle electrode was explanted 4 years after the implantation. The time between implantation and explantation did not significantly affect the complication rate.

The complication rate was not affected whether a resident under supervision performed the explantation or the explantation was performed by a qualified neurosurgeon (*P* = .564) nor if the operation was performed as an elective or emergency surgery (*P* = .531). On average, surgeries followed by complications lasted 20.12 minutes longer. The observation was not statistically significant (*P* = .142) (Table 6).

**TABLE 6.** Operation Characteristics^a^

Variable	Total sample n (%) or mean (SD)	No complications n (%) or mean (SD)	Complications n (%) or mean (SD)	*P* value
	n = 130	n = 106	n = 24	
Time between implantation and explantation in years	4.02 (4.32)	4.18 (4.28)	3.35 (4.52)	.33
Surgeon	n = 130	n = 106	n = 24	.564
Neurosurgeon	106 (81.5%)	85 (80.2%)	21 (19.8%)	
Resident (under supervision)	24 (18.5%)	21 (87.5%)	3 (12.5%)	
Type	n = 122	n = 99	n = 23	.531
Emergency	20 (16.4%)	15 (75.0%)	5 (25.0%)	
Elective	102 (83.6%)	84 (82.4%)	18 (17.6%)	
	n = 128	n = 105	n = 23	
Duration of operation	140 (77.0)	136.14 (79.74)	156.26 (62.14)	.142
	n = 131	n = 107	n = 24	
Postoperative hospitalization days	2.35 (2.39)	2.22 (2.44)	2.92 (2.10)	.011^b^

a Total sample size for different variables differs due to incomplete documentation available.

b Significant result.

On average, hospitalization time for patients undergoing SCS explantation was 2.35 days (SD = 2.39). Patients who suffered complications were hospitalized on average 1 day longer (2.22 vs 2.92, *P* = .011).

### Postoperative Pain Management

Patients who suffer from chronic pain are susceptible to pain management issues. For example, patients suffering from CRPS type I have previously demonstrated functional disturbances in processing pain.^13^ Owing to their subjective nature, abnormal pain levels were not counted as complications but were analyzed in the overall management of the patient. Experience of abnormal pain was divided into acute onset—within 3 weeks of operation, and late onset—from 3 weeks to 3 months postoperatively.

Patients experienced abnormal levels of pain after 16 (12.21%) operations. Patients reported disturbing incision site pain after 14 (10.69%) operations; in 8 cases—acute onset and in 6—late onset. Acute onset incision site pain prolonged initial hospitalization on average by 1 day (3.63 days vs 2.27 days, *P* = .12). Two patients required rehospitalization for pain treatment. In 3 patients, the pain became chronic. Further analysis will be presented in another publication.

## DISCUSSION

SCS explantation is a common neurosurgical procedure.^6,14^ The explantation rate of surgically implanted SCS paddle electrodes is 9%–25%, and the overall explantation rate for all types of SCS electrodes is up to 38%.^2,15-17^ Despite this, there are few reports examining the complication rate after SCS explantation, with most studies being case reports.^18-20^

Maldonado-Naranjo et al^19^ reported a complication rate of 12%, mainly consisting of minor complications, and major complications represented only 3% of the cases. In our study, the complication rate was higher at 18.32%. We can speculate that our study found a higher complication rate in part due to the inclusion of “subjective” complications such as paresthesia or abdominal pain that presented after the procedure without any clear explanation or radiological findings. The major complications rate was similar, representing 3.82%.

This study supports the concept that SCS treatment is reversible and explantation carries an acceptable complication rate. However, patients who had their stimulator explanted 3 times or more suffered significantly more complications than those who had their stimulation explanted 1 or 2 times (15.4% vs 62.5%, *P* = .005). We hypothesized that having the electrode removed from the same wound multiple times might predispose to more complications due to the formation of scar tissue, more difficult dissection, and poorer wound healing. Caution and careful patient selection are advised when considering multiple re-explantations.

Our study supports the findings by Hoelzer et al and Harland et al that revision operations are generally safe.^21,22^ Our study showed a similar complication rate between replacement/relocation and simple explant. However, it is worth noticing that 2 of our major complications (patients developing paraparesis after the operation) occurred in patients having their electrodes replaced. Although rare, the occurrence of major complications such as epidural hematoma supports the notion that SCS explantations should be performed at a tertiary medical facility where competence and resources are available to deal with acute, severe complications in a timely manner.

Two major complications occurred when SCS was removed due to infection (patients developed an abscess after the explantation requiring a new operation). Special attention should be paid to these patients. Treatment with antibiotics guided by bacterial cultures and a careful follow-up is advised.

Smoking and DM are known factors to increase surgical complication rates.^23-25^ Smoking has been associated with increased revisions due to lead migration or new pain symptoms, leading to failure in treatment.^26,27^ In our study, smoking significantly increased the complication rate (*P* = .023). Previous studies have shown a reduction in postoperative complications when patients quit smoking at least 1 year before their neurosurgical operation.^4,11,12^ Patients with DM had as well higher complication rates, although this finding was not statistically significant. Quitting smoking and careful management of DM preoperatively should be of the utmost importance.

Like Maldonado-Naranjo et al, we documented a higher complication rate in the cervical electrodes' explantations than the thoracic (25.7% vs 16.1%, *P* = .207).^19^ Neither study could prove statistical significance.

### Postoperative Pain Management

Postoperative abnormal pain levels can severely affect a patient's mental and physical well-being. In our data, after 12.21% of SCS explantations, patients experienced abnormal pain levels, leading to prolonged hospitalizations and, in some cases, to the development of a new area of chronic pain. In this group of patients, abnormal pain levels are more common and thus should be discussed with the patients before the procedure.^13^

### Limitations

One of our main limitations is that this is a retrospective study, where improper documentation can hamper data collection.

## CONCLUSION

To the best of our knowledge, this is the largest report to describe the postoperative complications related to SCS paddle electrode explantation. This study supports the idea that SCS explantation can be considered a safe operation with an acceptable postoperative complication rate and can serve as a guideline for future patient consultations when considering SCS paddle electrode explantation or revision.
